# Congenital pulmonary airway malformation mimicking lung cancer: A case report

**DOI:** 10.1097/MD.0000000000016057

**Published:** 2019-06-14

**Authors:** Ying Zhao, Yongxiang Zhang, Qi Leng, Zhenwu Li, Peng Pang, Xiaoli Qi

**Affiliations:** a Department of Respiratory; b Department of Radiology; c Department of Thoracic Surgery; d Department of Pathology, Capital Medical University Daxing Teaching Hospital, Beijing, China.

**Keywords:** congenital cystic adenomatoid malformation, congenital pulmonary airway malformation, lung cancer, misdiagnosis

## Abstract

**Rationale:**

Congenital pulmonary airway malformation (CPAM) is a rare developmental deformity of the lower respiratory tract. The disease occurs more in newborns. However, on rare occasions, CPAM can be found in adults. Radiologic features of CPAM include cystic or solid mass pattern. In an elderly patient, CPAM can be easily misdiagnosed as lung cancer.

**Patient concerns:**

A 66-year old woman was admitted with complaints of chronic cough, expectoration. Her past history was unremarkable with no history of tuberculosis or smoking. Physical examination was normal. Computerized tomography of the chest showed an irregular cystic lesion in right lower lobe.

**Diagnosis:**

Histopathological results confirmed the diagnosis of CPAM.

**Intervention:**

The right pulmonary wedge resection was performed via thoracoscopic surgery.

**Outcomes:**

On follow up 1 year later, the patient is asymptomatic.

**Lessons:**

CPAM is rare in adults, and imaging cannot accurately distinguish CPAM from thin-walled cystic lung cancer. Hence, histopathology is mandatory to confirm the diagnosis.

## 1 Introduction

Congenital pulmonary airway malformation (CPAM) is a rare developmental deformity of the lower respiratory tract.^[1–3]^ Imaging examination is an important way of diagnosing CPAM, which is characterized by bronchiolar adenomatous hyperplasia and cyst formation. The disease is often found in newborns. However, on rare occasions, it can be found in adults. A common complication of CPAM is recurrent pulmonary infection. It may be diagnosed in adults because of complications or suspected malignancy.^[4,5]^ So it is rarely discovered in asymptomatic adults, the preoperative diagnosis is challenging because congenital cystic adenomatoid malformations (CCAM) can be confused with other more common lesions. Therefore, we report a case of a 66-year-old woman with cystic lesion in the right lower lobe which was highly suspicious of lung cancer. Surgical resection of the lesion followed by histopathological confirmed a CPAM.

## 2 Case report

**Figure 1:**
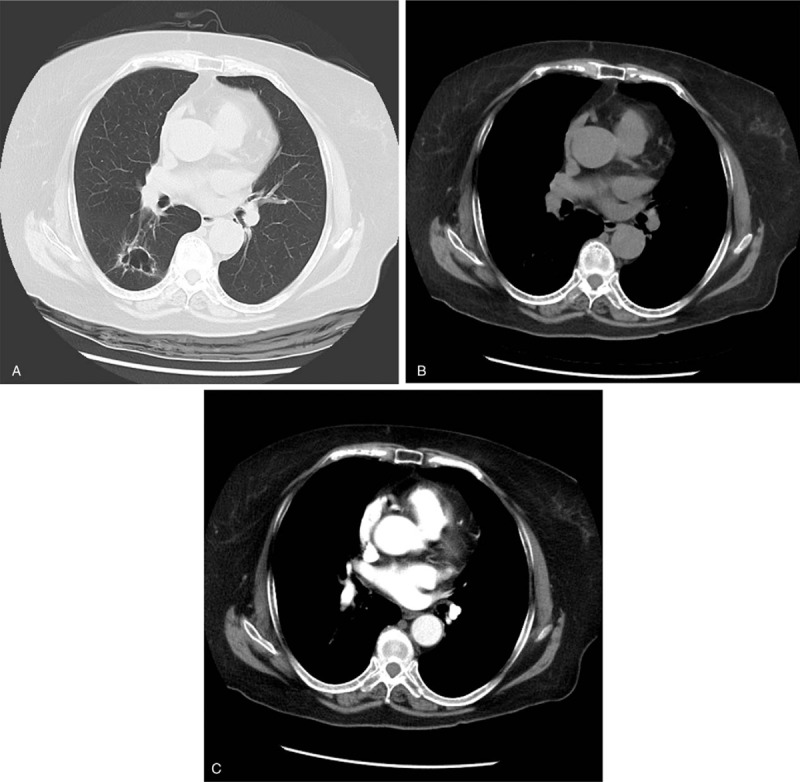
(A–C) Chest radiography showed a 35 × 22 mm cystic lesion in the right lower lung. Lung window setting (A), soft tissue window setting (B), and contrast enhanced chest CT (C). CT = computerized tomography.

**Figure 2:**
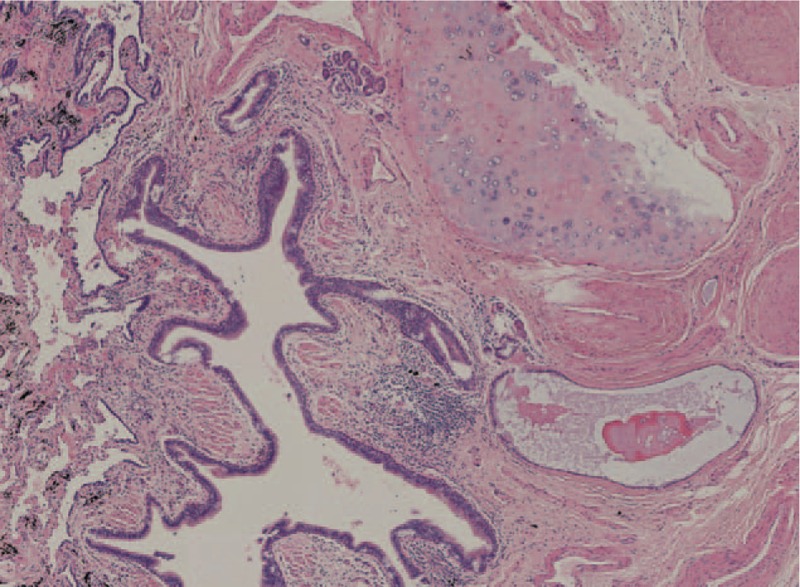
Histopathology (H&E, ×40), lesion composed of varying sized cysts, lined by pseudostratified ciliated columnar epithelium. The wall of the cysts contains smooth muscle, blood vessels, and cartilage. Relatively normal alveoli may be seen interspersed between the cyst.

A 66-year old Chinese woman, born in Beijing, was admitted with complaints of cough, expectoration since for 3 months. She denied fever, night sweats, loss of weight, chest pain, or hemoptysis. Her past history was unremarkable with no history of tuberculosis or smoking. Physical examination was normal. Clinically, electrocardiography and routine laboratory tests were normal. Contrast-enhanced computerized tomography of the chest showed 35 × 22 mm irregular cystic lesion in right lower lobe, wall thickness of 0.5 mm to 3.5 mm, smooth inner margin, septum was seen within the cyst. The lesion also showed mild distortion of surrounding lung tissue with no evidence of abnormal arterial blood supply (Fig. 1A–C). The result of brush test showed cells with heteromorphic nucleus by bronchoscopy. Right lower lobe thoracoscopic wedge resection was done. The size of the lung tissue removed was 10.0 × 2.5 × 1.5 cm. Histopathology (Fig. 2) showed cystic tumor of 4.5 × 2.0 × 1.5 cm, composed of multiple varying sized cysts lined by pseudostratified ciliated columnar epithelium with few areas of necrosis. All these features were consistent with the diagnosis of type 1 CPAM. On follow up 1 year later, the patient is asymptomatic.

## 3 Discussion

CPAM, formerly known as CCAM, was first described by Ch’in and Tang in 1949. The pathogenesis is not clear but segmental bronchial atresia has been attributed as the primary pathogenetic defect that results in the development of a CCAM distal to the defect.^[1]^ Incidence of CCAM of the lung was found to be 0.0029% to 0.004% in newborns.^[6,7]^ However, on rare occasions, it can be found in adults.

In histopathology, CPAM is a hamartoma-like lesion. Stocker has described 3 types of CCAM classification on the basis of histopathologic appearance in 1977.^[8]^ Type 1 is thought to originate in the distal or proximal bronchioles. Type 1, the most frequently occurring type (60%–70%), was a lesion consisting of single or multiple large cysts (more than 2 cm in diameter) lined by ciliated pseudostratified columnar epithelium. The walls of the cysts contain prominent smooth muscle and elastic tissue. Mucus-producing cells are present in 32% of the cases, and cartilage in the wall was seen in 5% to 10%. Relatively normal alveoli may be seen between the cysts. Ninety-five percent of type 1 patients have only 1 lung lobe involved.^[9]^ Type 2 was composed of multiple small cysts (less than 1 cm in diameter) lined by ciliated cuboidal to columnar epithelium and was associated with other anomalies. There were no mucous cells or cartilage. Type 3 was composed of regularly spaced bronchiole-like structures separated by masses of cuboidal epithelium-lined alveolus-like structures. In 2002, Stocker added type 0 and type 4 to 3 types of CCAM classification, both of which were very rare. Meanwhile, CCAM was renamed CPAM. Each type of CPAM has unique pathological features.^[10]^ The clinical manifestations of CPAM mainly depend on the lesion of the size, number, location, and compression of surrounding tissues. In newborns, most of them presenting clinical symptoms may include fever, cough, progressive dyspnea, cyanosis, or hemoptysis. In adults, presenting clinical symptoms may also include cough, expectoration, chest tightness, and dyspnea. CPAM are often found when they cause recurrent pulmonary infection.^[4,5]^ Clinical symptoms are not obvious when the lesion is confined to a single lobe or lung segment. Imaging examination is an important way of CPAM, which is characterized by bronchiolar adenomatous hyperplasia and cyst formation. Its imaging pattern is described into 3 types:

(1) single or multicystic pattern,
(2) nonhomogeneous mass consistent with the gross configuration of numerous evenly spaced cysts usually less than 1 cm size,
(3) a cystic or solid mass.^[11]^

Radiologic features of type 1 CPAM include involvement of single lobe, predominantly lower lung fields with left lung more than right.^[12]^ Final diagnosis and classification required pathological confirmation. It is not clear why this case has gone undetected. We hypothesized 3 possible causes: first, the lack of symptoms of recurrent respiratory infections; second, the limitation of imaging lesions; and third, the absence of annual routine chest imaging examination. These may explain that the patient was not detected early. In our case, the patient was elderly female presented in 6th decade, with no history of recurrent pulmonary infection. Her computerized tomography (CT) chest showed irregular cystic lesion in peripheral right lower lobe, with uneven wall thickness and septum within the cyst. These features raised possibility of malignancy. Therefore, it needs to be differentiated from lung cancer. There is a kind of lung cancer presenting as thin-walled cyst or cavity has been described in various case reports.^[13–17]^ It usually presents as peripheral lung lesion, most of them were adenocarcinomas. These are more common in females than male. CT features suggestive of malignancy include uneven thickening of the thin-walled cavity, irregular margin, septum in cavity, nodule formation, presence of short spicules, blood vessel convergence signs, signs of lobulation or pleural indentation. Most important is increasing size of cyst or cavity with thickening of its wall. Histopathology shows that carcinoma cells grow randomly along the cyst wall.^[15–17]^ Hence, the need for surgical resection and pathological examination is reaffirmed. However, in our case, diagnosis of type 1 CPAM was confirmed with histopathology with no evidence of malignancy. Type 1 CPAM has good prognosis; however, a risk of malignant transformation was reported to have an association with a mucinous bronchioloalveolar carcinoma.^[18–20]^ Currently, lobectomy is considered to be the best method for treating it.^[18]^

We report a unique case of pulmonary cystic lesion masquerading pulmonary malignancy, diagnosed as CPAM in patient presenting unusually in 6th decade. It poses a diagnostic challenge, broadens the thinking of clinicians and increases the understanding of CPAM. Though meticulous imaging may help to differentiate it from malignancy, histopathology is mandatory to confirm the diagnosis.

## Author contributions

**Data curation:** Qi Leng, Zhenwu Li, Peng Pang.

**Formal analysis:** Zhenwu Li, Peng Pang, Xiaoli Qi.

**Resources:** Qi Leng.

**Supervision:** Ying Zhao.

**Validation:** Qi Leng, Zhenwu Li.

**Writing – original draft:** Ying Zhao, Xiaoli Qi.

**Writing – review and editing:** Ying Zhao, Yongxiang Zhang, Xiaoli Qi.
